# Mental Health, Suicidality, and Connectedness Among High School Students During the COVID-19 Pandemic — Adolescent Behaviors and Experiences Survey, United States, January–June 2021

**DOI:** 10.15585/mmwr.su7103a3

**Published:** 2022-04-01

**Authors:** Sherry Everett Jones, Kathleen A. Ethier, Marci Hertz, Sarah DeGue, Vi Donna Le, Jemekia Thornton, Connie Lim, Patricia J Dittus, Sindhura Geda

**Affiliations:** ^1^Division of Adolescent and School Health, National Center for HIV, Viral Hepatitis, STD, and TB Prevention, CDC; ^2^Division of Violence Prevention, National Center for Injury Prevention and Control, CDC; ^3^ICF International, Rockville, Maryland

## Abstract

Disruptions and consequences related to the COVID-19 pandemic, including school closures, social isolation, family economic hardship, family loss or illness, and reduced access to health care, raise concerns about their effects on the mental health and well-being of youths. This report uses data from the 2021 Adolescent Behaviors and Experiences Survey, an online survey of a probability-based, nationally representative sample of U.S. public- and private-school students in grades 9–12 (N = 7,705), to assess U.S. high school students’ mental health and suicidality during the COVID-19 pandemic. The study also examines whether mental health and suicidality are associated with feeling close to persons at school and being virtually connected to others during the pandemic. Overall, 37.1% of students experienced poor mental health during the pandemic, and 31.1% experienced poor mental health during the preceding 30 days. In addition, during the 12 months before the survey, 44.2% experienced persistent feelings of sadness or hopelessness, 19.9% had seriously considered attempting suicide, and 9.0% had attempted suicide. Compared with those who did not feel close to persons at school, students who felt close to persons at school had a significantly lower prevalence of poor mental health during the pandemic (28.4% versus 45.2%) and during the past 30 days (23.5% versus 37.8%), persistent feelings of sadness or hopelessness (35.4% versus 52.9%), having seriously considered attempting suicide (14.0% versus 25.6%), and having attempted suicide (5.8% versus 11.9%). The same pattern was observed among students who were virtually connected to others during the pandemic (i.e., with family, friends, or other groups by using a computer, telephone, or other device) versus those who were not. Comprehensive strategies that improve feelings of connectedness with others in the family, in the community, and at school might foster improved mental health among youths during and after the COVID-19 pandemic.

## Introduction

Emerging data suggest that the COVID-19 pandemic has negatively affected the mental health of many children and adolescents (*1*). Before the pandemic, youth mental health was already an important public health concern (*2*,*3*). For example, among high school students nationwide, significant increases occurred between 2009 and 2019 in having persistent feelings of sadness or hopelessness (26.1% to 36.7%), having seriously considered attempting suicide (13.8% to 18.8%), and having attempted suicide (6.3% to 8.9%) (*2*). For many youths during the pandemic, mental health was affected by school closures, social isolation, family economic hardship, fear of family loss or illness, and reduced access to health care because of inadequate insurance coverage or medical office closures and reduced hours (*1*). Two longitudinal studies on adolescent mental health during the pandemic found increases in depression and anxiety over the course of the pandemic (*4*,*5*). In one study, these symptoms were predicted by COVID-19–related worries, online learning difficulties, and increased conflict with parents (*4*). In another study, emergency department visits for suspected suicide were 50.6% higher among girls and 3.7% higher among boys from February through March 2021 than during the same period in 2019 (*6*). To understand the impact of COVID-19 on youth mental health and to identify potential protective factors, this study examines U.S. high school students’ mental health and suicidality during the COVID-19 pandemic, including the relation between mental health and connectedness to school, family, friends, and community groups. Public health and health care professionals, communities, schools, families, and adolescents can use these findings to better understand students’ mental health and suicidal thoughts and attempts during the pandemic and how fostering connectedness at school and with others could be one strategy to promote adolescent health and well-being during the pandemic and beyond.

## Methods

### Data Source

This report includes data from the Adolescent Behaviors and Experiences Survey (ABES), which was conducted by CDC during January–June 2021 to assess student behaviors and experiences during the COVID-19 pandemic. ABES was a one-time, probability-based online survey of U.S. high school students. ABES used a stratified, three-stage cluster sampling approach to obtain a nationally representative sample of public- and private-school students in grades 9–12 in the 50 U.S. states and the District of Columbia (N = 7,705). Participation in ABES was voluntary; each school and teacher decided whether students completed the survey during instructional time or on their own time. Additional information about ABES sampling, data collection, response rates, and processing is available in the overview report of this supplement (*7*). The ABES questionnaire, datasets, and documentation are available at https://www.cdc.gov/healthyyouth/data/abes.htm.

### Measures

This analysis included seven measures: 1) poor mental health during the pandemic, 2) poor mental health during the past 30 days, 3) persistent feelings of sadness or hopelessness during the past 12 months, 4) serious consideration of attempting suicide during the past year, 5) attempted suicide during the past year, 6) feeling close to persons at school (time frame not specified), and 7) being virtually connected to others during the pandemic (Table 1). For the pandemic-related questions, the time frame was not further specified. In addition, the following demographic characteristics were analyzed: sex, sexual identity (heterosexual; gay, lesbian, or bisexual; or other or questioning), and race and ethnicity (non-Hispanic American Indian or Alaska Native [AI/AN], non-Hispanic Asian [Asian], non-Hispanic Black [Black], Hispanic or Latino [Hispanic], non-Hispanic persons of multiple races [multiracial], non-Hispanic Native Hawaiian or other Pacific Islander, and non-Hispanic White [White]).

**TABLE 1:** Question and analytic coding for health behaviors and experiences, by variable assessed — Adolescent Behaviors and Experiences Survey, United States, January–June 2021

Variable	Question	Analytic coding
Poor mental health during the pandemic	During the COVID-19 pandemic, how often was your mental health not good? (Poor mental health includes stress, anxiety, and depression.)	Always or most of the time versus never, rarely, or sometimes
Poor mental health during the past 30 days	During the past 30 days, how often was your mental health not good? (Poor mental health includes stress, anxiety, and depression.)	Always or most of the time versus never, rarely, or sometimes
Persistent feelings of sadness or hopelessness	During the past 12 months, did you ever feel so sad or hopeless almost every day for two weeks or more in a row that you stopped doing some usual activities?	Yes versus no
Seriously considered attempting suicide	During the past 12 months, did you ever seriously consider attempting suicide?	Yes versus no
Attempted suicide	During the past 12 months, how many times did you actually attempt suicide?	≥1 time versus 0 times
Felt close to persons at school	Do you agree or disagree that you feel close to people at your school?	Strongly agree or agree versus not sure, disagree, or strongly disagree
Virtually connected to others during the pandemic	During the COVID-19 pandemic, how often were you able to spend time with family, friends, or other groups, such as clubs or religious groups, by using a computer, phone, or other device? (Do not count attending school online.)	Always, most of the time, or sometimes versus never or rarely

### Analysis

Weighted prevalence estimates and 95% CIs were calculated for all study variables among students overall and by demographic characteristics. Statistically significant pairwise differences for the study variables by demographic characteristics, and for associations between mental health, suicidality, and connectedness, were determined by *t*-tests for proportions. Analyses were completed using SUDAAN (version 11.0.3; RTI International) to account for the complex survey design and weighting. Differences were considered statistically significant if the p value was <0.05. Only significant results are presented in the text. 

## Results

### Poor Mental Health

Approximately one in three high school students experienced poor mental health (most of the time or always) during the COVID-19 pandemic (37.1%) and during the past 30 days (31.1%) (Table 2). During the 12 months before the survey, 44.2% experienced persistent feelings of sadness or hopelessness; that is, had ever felt so sad or hopeless almost every day for two weeks or more in a row that they stopped doing some usual activities.

**TABLE 2:** Percentage of students with poor mental health, persistent feelings of sadness or hopelessness, suicidal thoughts and attempts, and who experienced connectedness,* by demographic characteristics — Adolescent Behaviors and Experiences Survey, United States, January–June 2021

Characteristic	Poor mental health during the pandemic	Poor mental health during the past 30 days	Persistent feelings of sadness or hopelessness	Seriously considered attempting suicide	Attempted suicide	Felt close to persons at school	Virtually connected to others during the pandemic
	%^†^ (95% CI)	%^†^ (95% CI)	%^†^ (95% CI)	%^†^ (95% CI)	%^†^ (95% CI)	%^†^ (95% CI)	%^†^ (95% CI)
**Sex**							
Female	48.9^§^ (45.6–52.3)	41.6^§^ (38.4–44.9)	56.5^§^ (53.4–59.5)	26.0^§^ (23.4–28.6)	12.4^§^ (10.5–14.5)	40.8^§^ (36.8–44.8)	71.8 (69.7–73.8)
Male	24.4 (22.3–26.7)	19.6 (17.6–21.8)	31.4 (29.1–33.7)	13.6 (12.0–15.4)	5.3 (4.2–6.6)	53.0 (50.7–55.4)	71.7 (69.4–74.0)
**Race and ethnicity**							
AI/AN, non-Hispanic	23.3^¶,^**^,††^ (15.8–33.0)	20.5 (9.0–40.2)	49.5^¶¶,^*** (42.2–56.9)	23.3 (15.6–33.5)	20.1^¶,††,¶¶,^*** (12.4–30.9)	50.9*** (39.4–62.3)	70.6 (46.0–87.1)
Asian, non-Hispanic	33.7 (27.5–40.5)	29.1 (23.7–35.1)	40.2** (34.4–46.3)	15.9^††^ (12.6–19.9)	7.4 (4.9–11.0)	44.3^††,^*** (38.2–50.6)	73.4 (67.1–78.9)
Black, non-Hispanic	28.0^¶,^**^,††^ (23.3–33.2)	25.6^¶,^**^,††^ (22.0–29.5)	39.7^¶,^** (35.9–43.6)	16.2**^,††^ (13.0–20.0)	10.0 (7.7–12.9)	33.5^¶,^**^,††^ (29.1–38.2)	68.9^††^ (65.3–72.3)
Hispanic or Latino	36.8 (33.2–40.6)	31.1 (27.9–34.6)	46.4 (42.1–50.8)	19.7 (16.9–22.7)	8.4 (6.5–10.7)	41.6**^,††^ (37.1–46.2)	67.2 (63.7–70.5)^††^
Multiracial, non-Hispanic	40.0 (32.8–47.7)	32.5 (27.0–38.5)	51.0 (44.5–57.4)^††^	25.6 (18.1–34.8)	12.3 (8.0–18.5)	50.8 (43.8–57.8)	68.7 (61.6–75.1)
NH/OPI, non-Hispanic	—^§§^	—	45.8 (19.2–75.0)	12.4 (3.3–36.5)	__	—	—
White, non-Hispanic	40.1 (37.4–42.9)	32.8 (29.6–36.2)	43.8 (40.3–47.2)	21.0 (18.6–23.6)	8.9 (7.1–11.0)	52.3 (49.5–55.1)	75.1 (73.2–76.9)
**Sexual identity**							
Heterosexual	30.3 (27.6–33.2)	25.5 (22.5–28.8)	36.7 (34.1–39.4)	13.6 (11.7–15.8)	5.2 (4.2–6.5)	50.1 (47.1–53.1)	72.7 (70.8–74.5)
Gay, lesbian, or bisexual	63.8^†††^ (58.5–68.8)	54.9^†††,§§§^ (49.5–60.2)	75.7^†††,§§§^ (70.9–79.9)	46.8^†††,§§§^ (41.5–52.2)	26.3^†††,§§§^ (21.8–31.4)	36.8^†††^ (32.2–41.6)	69.9 (65.1–74.2)
Other or questioning	61.5^†††^ (54.6–67.9)	45.7^†††^ (40.5–50.9)	68.7^†††^ (63.6–73.4)	39.5^†††^ (34.6–44.7)	16.5^†††^ (11.8–22.7)	33.6^†††^ (29.1–38.4)	69.6 (65.6–73.3)
**Total**	**37.1 (34.6–39.6)**	**31.1 (28.5–33.7)**	**44.2 (41.6–46.8)**	**19.9 (18.0–22.0)**	**9.0 (7.7–10.5)**	**46.6 (44.1–49.2)**	**71.8 (70.2–73.3)**

**Abbreviations:** AI/AN = American Indian or Alaska Native; NH/OPI = Native Hawaiian or other Pacific Islander.

* Refer to Table 1 for variable definitions.

^†^ All percentages are weighted.

^§^ Significantly different from male students, based on *t*-test analysis (p<0.05).

^¶^ Significantly different from Hispanic students, based on *t*-test analysis (p<0.05).

** Significantly different from non-Hispanic multiracial students, based on *t*-test analysis (p<0.05).

^††^ Significantly different from non-Hispanic White students, based on *t*-test analysis (p<0.05).

^§§^ Results suppressed because n<30.

^¶¶^ Significantly different from non-Hispanic Asian students, based on *t*-test analysis (p<0.05).

*** Significantly different from non-Hispanic Black students, based on *t*-test analysis (p<0.05).

^†††^ Significantly different from heterosexual students, based on *t*-test analysis (p<0.05).

^§§§^ Significantly different from other or questioning students based on *t*-test analysis (p<0.05).

The prevalence of poor mental health during the pandemic, poor mental health during the past 30 days and persistent feelings of sadness or hopelessness were higher among female than male students (Table 2). Although differences by race and ethnicity were detected for each of these three variables, no consistent patterns were found. The prevalence of poor mental health during the pandemic was higher among gay, lesbian, or bisexual students and other or questioning students than among heterosexual students. The prevalence of poor mental health during the past 30 days and of persistent feelings of sadness or hopelessness was highest among gay, lesbian, or bisexual students, followed by other or questioning students. Heterosexual students had the lowest prevalence.

### Suicidal Thoughts and Behaviors

During the 12 months before the survey, 19.9% of students had seriously considered attempting suicide, and 9.0% had attempted suicide. The prevalence of having seriously considered attempting suicide and attempting suicide was higher among female students than male students and varied by race and ethnicity. The prevalence of having seriously considered attempting suicide was higher among White students than Black or Asian students and higher among multiracial students than Black students. The prevalence of having attempted suicide was higher among AI/AN students than White, Black, Hispanic, or Asian students. The prevalence of having seriously considered attempting suicide and attempted suicide was highest among gay, lesbian, or bisexual students, followed by other or questioning students. Heterosexual students had the lowest prevalence.

### Connectedness

At the time of the survey, 46.6% of students strongly agreed or agreed that they felt close to persons at school. In contrast, 71.8% of students sometimes, most of the time, or always spent time virtually (i.e., by using a computer, telephone, or other device) with family, friends, or others during the pandemic. The prevalence of feeling close to persons at school was higher among male students than female students. Being virtually connected to others during the pandemic did not vary by sex. The prevalence of feeling close to persons at school and being virtually connected to others varied by race and ethnicity. The prevalence of feeling close to persons at school was higher among White students than Black, Hispanic, and Asian students; higher among Hispanic, Asian, AI/AN, and multiracial students than Black students; and higher among multiracial students than Hispanic students. The prevalence of being virtually connected to others was higher among White students than Black and Hispanic students. The prevalence of feeling close to persons at school was higher among heterosexual students than gay, lesbian, or bisexual students and other or questioning students; however, being virtually connected to others during the pandemic did not vary by sexual identity.

### Connectedness and Mental Health

Compared with those who did not feel close to persons at school, students who felt close to persons at school had a lower prevalence of poor mental health during the pandemic (28.4% versus 45.2%) and during the past 30 days (23.5% versus 37.8%), of persistent feelings of sadness or hopelessness (35.4% versus 52.9%), of having seriously considered attempting suicide (14.0% versus 25.6%), and of having attempted suicide (5.8% versus 11.9%) (Figure). Similarly, students who were virtually connected to others during the pandemic had a lower prevalence of poor mental health during the pandemic (35.5% versus 42.0%) and during the past 30 days (28.7% versus 36.8%), of persistent feelings of sadness or hopelessness (41.9% versus 51.7%), of having seriously considered attempting suicide (18.4 versus 24.9%), and of having attempted suicide (8.0% versus 12.2%) compared with those who were not virtually connected to others during the pandemic.

**FIGURE:**
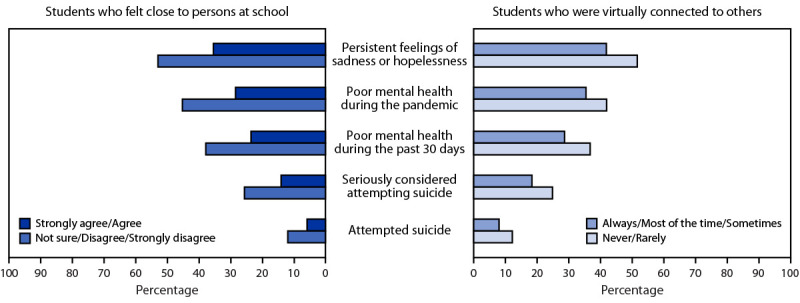
Persistent feelings of sadness or hopelessness, perceptions of mental health, and suicidal thoughts and attempts among high school students during the COVID-19 pandemic, by feeling close to persons at school* and being virtually connected^†^ — Adolescent Behaviors and Experiences Survey, United States, January–June 2021 * All comparisons of having felt close versus not sure, disagree, or strongly disagree they felt close were significantly different, based on *t*-test analysis (p<0.05). ^†^ All comparisons of being connected versus never or rarely felt connected were significantly different, based on *t*-test analysis (p<0.05).

## Discussion

More than one in three high school students (37.1%) experienced poor mental health during the COVID-19 pandemic. In addition, 44.2% of students experienced persistent feelings of sadness or hopelessness, almost 20% seriously considered suicide, and 9.0% attempted suicide during the 12 months before the survey. The prevalence of poor mental health and suicidality was high across students of all sex, sexual identity, and racial and ethnic groups; however, poor mental health, persistent feelings of sadness or hopelessness, and suicidal thoughts and behaviors were less prevalent among those who felt close to persons at school and were virtually connected with others during the pandemic.

During the COVID-19 pandemic, students’ feelings of being connected to school were likely reduced by extensive school closures and transitions to virtual learning (*8*). Efforts to improve connectedness to schools, peers, and family are critical to protecting the mental health and well-being of youths (*9*), particularly in the context of ongoing pandemic-related stressors. Evidence from previous outbreaks suggests that the pandemic might have long-term consequences for youth mental health and well-being and be associated with potential increases in youth depression, anxiety, and post-traumatic stress disorder, which underscores the urgent need to address mental health needs among youths (*10*).

In addition to providing youths with access to needed mental health care (*11*), comprehensive approaches that promote help-seeking behaviors, connections to trusted adults and supportive peers, and engagement in community activities have been shown to have many benefits including improved feelings of connectedness, better mental health, reduced risk for suicide, reduced prevalence of health risk behaviors, and better academic achievement (*9*,*12*). Positive experiences during childhood, including school connectedness, can build resilience and protect or buffer adults who have experienced multiple childhood traumas (*13*).

To foster school connectedness and promote positive school climates, school districts can implement schoolwide programs such as those focused on social and emotional learning, professional development for staff to improve classroom management, and strategies to foster relationships between students, their families, and school staff. Another way to foster school connectedness and promote positive school climates is for school districts to analyze school disciplinary policies to ensure they are being implemented equitably across racial and ethnic groups (*9*,*14*,*15*). In addition to engaging with their child’s school, parents and caregivers can build relationships with their child through open discussions and shared activities (*15*).

## Limitations

General limitations to ABES are outlined in the overview report in this supplement (*7*). The findings in this report are subject to at least four specific limitations. First, the mental health and suicidality variables used in this study are important indicators of students’ mental well-being; however, the questions were not designed to diagnose clinical depression. Second, most students were virtually connected to others, such as family, friends, or other groups, during the pandemic. Among students who were never or rarely virtually connected, it is unknown if that was a function of more in-person interactions; individual choice; a lack of family, friends, or other groups with whom students could be connected; or a lack of access to the technology needed by the student or others with whom the student would connect. Third, the survey did not ask students to indicate whether, at the time of the survey or in weeks or months preceding the survey, they attended school in person, remotely, or both in person and remotely. Students’ method of attendance might be a confounder for the findings related to students’ feeling of connectedness. Finally, because this was a one-time survey, no longitudinal data from studies using the same data collection methods are available to directly compare pre- and postpandemic mental health status among youths.

## Conclusion

Mental health issues among youths are an important public health concern during the ongoing COVID-19 pandemic. However, the findings in this report also indicate that poor mental health, persistent feelings of sadness or hopelessness, and suicidal thoughts and behaviors were less prevalent among those who felt close to persons at school and were virtually connected with others during the pandemic. Comprehensive strategies that improve connections with others at home, in the community, and at school might foster improved mental health among youths during and after the pandemic.
